# Genome Sequence of *Leuconostoc citreum* DmW_111, Isolated from Wild *Drosophila*

**DOI:** 10.1128/genomeA.00507-17

**Published:** 2017-06-15

**Authors:** Sage M. Wright, Courtney Carroll, Amber Walters, Peter D. Newell, John M. Chaston

**Affiliations:** a Department of Biology, Brigham Young University, Provo, Utah, USA; b Department of Plant and Wildlife Sciences, Brigham Young University, Provo, Utah, USA; c Department of Biological Sciences, SUNY Oswego, Oswego, New York, USA

## Abstract

Isolates of the lactic acid bacterium *Leuconostoc citreum* are a major part of fermentation processes, especially in Korean kimchi. Here, we present the genome of *L. citreum* DmW_111, isolated from wild *Drosophila melanogaster*; analysis of this genome will expand the diversity of genome sequences for non-*Lactobacillus* spp. isolated from *D. melanogaster*.

## GENOME ANNOUNCEMENT

*Leuconostoc citreum* plays a significant role in the fermentation of many foods (1). As a major component of the microflora found in kimchi, a popular Korean dish (1–3), it is the primary species in the early and mid-stages of kimchi fermentation and ripening (2). Strains also have been identified in sourdough bread (4). Typically producing a lemon-colored pigment that is its namesake, *L. citreum* is resistant to the antibiotic vancomycin (5). Species of *Leuconostoc* have been detected in association with the fruit fly *Drosophila melanogaster*, whose diet can contain communities of fermentation microbes; for example, in one study the *Leuconostoc* genus represented 3% of the fly’s microbiome (6). To better understand the genomics of *Drosophila-*associated lactic acid bacteria communities, we sequenced the genome of an *L. citreum* strain isolated from wild *Drosophila*.

Wild *Drosophila* samples were collected from a household kitchen in Ithaca, New York, USA (42.427481 N, 76.463983 W). The flies were homogenized and diluted on modified MRS medium (7). A single colony was streaked for isolation, identified as *L. citreum* by Sanger sequencing of the 16S rRNA gene, and designated strain DmW_111. The strain was cultured for 2 days in the modified MRS medium, and DNA was extracted from <10^9^ cells using the Qiagen DNeasy blood and tissue kit. DNA was fragmented using NEBNext dsDNA Fragmentase, and fragments were prepared for adapter ligation and ligated using components of the NEBNext Ultra II DNA library prep kit. The library size was selected for 850 bp using Ampure XP magnetic beads; library concentration was quantified on a Qubit version 2.0 fluorometer. We performed 2 × 250 sequencing of the library on an Illumina HiSeq2500. A total of 4,865,277 paired-end reads passed quality filtering and were assembled *de novo* using Velvet version 1.2.10 (8), as we have previously described (9). Reads were randomly split into eight different bins, each representing around 200× genome coverage, and each bin was assembled into a separate genome after empirically determining the ideal *k*-mer length using the max *N*_50_ values. The best assemblies for each bin were assembled to create the assembly of the complete genome. The final assembly used a *k*-mer length of 217 bp. The total genome size was 1.825 Mb and consisted of 20 contigs, with an *N*_50_ of 315,566 bp. The max contig length was 641,482 bp; 1,852 genes were annotated using the NCBI genome submission pipeline. We confirmed the taxonomic assignment as *L. citreum* using ANIm analysis in JSpeciesWS (10).

Preliminary analysis using annotations created in RAST (11) revealed that our strain is missing genes for histidine biosynthesis when compared to *L. mesenteroides* strain ATCC 8293. However, our strain has genes for adhesion and sugar alcohols that are missing in *L. mesenteroides*. These differences present a starting point for future research to begin to understand the possible interactions between *L. citreum* and *D. melanogaster*.

### Accession number(s).

This whole-genome shotgun project has been deposited in GenBank under the accession no. NDXG00000000. The version described in this paper is the first version, NDXG01000000.
